# A Valuable Substitute for Ergot

**Published:** 1853-04

**Authors:** B. H. Washington

**Affiliations:** Woodbury


From the Nashville Jour. Mec. and Surg.

*A Valuable Substitute for Ergot.* By Dr. B. H. Washington, of Woodbury,

Dr. Washington has recently discovered that dry-cupping, applied to the spinal origin of nerves, has the effect of stimulating the function of the organ to which the nerves are distributed. Applied to the lowest part of the sacrum, dilatation of the os uteri is produced; and applied higher up, contraction of the uterus follows.

In an obstetrical case, where the pains had endured fourteen hours without producing any perceptible effect, in consequence of rigidity of the os uteri, Dr. Washington applied a dry-cup as low down on the sacrum as possible, so as to cover the origin of the nerves to the os uteri. The result was most satisfactory, for complete relaxation ensued; at the next pain the head descended to the outlet, and at the second pain the patient was safely delivered, and that in less than ten minutes from the application of the cups. In tedious labor, the cup should be applied, first, to the lowest point of the sacrum, and if, in the course of ten or fifteen minutes, the patient is not delivered, another should be applied higher up, so as to cause the uterus to contract. *The lower one should always be on when the upper one is applied*, so as to insure relaxation of the os uteri when the pains come on.

In cases of retained placenta, a contrary course is recommended: the cups are to be applied higher up, so as to cause the uterus to contract at once, the relaxation of the os uteri being always sufficient after the fœtus has passed.

The great advantage of this method of causing the os uteri to relax, and the uterus to contract, over the plan of giving ergot, needs no other recommendation than a simple statement of facts. When ergot is administered, the woman is delivered by main force, in opposition to the usual proceedings of nature, without any relaxation except that produced by the most fearful pains. By dry-cupping such a complete relaxation is produced, that two or three pains are sufficient, and the amount of suffering is not more than ordinary.

